# Successful closure of gastric wall defect after endoscopic full-thickness resection using novel anchor pronged clips: a case report

**DOI:** 10.1055/a-2209-0076

**Published:** 2023-12-11

**Authors:** Hiroya Mizutani, Yosuke Tsuji, Hiroyuki Hisada, Yoshiyuki Miwa, Koichi Yagi, Yasuyuki Seto, Mitsuhiro Fujishiro

**Affiliations:** 1 13143 Department of Gastroenterology/Department of Next-Generation Endoscopic Computer Vision, Graduate School of Medicine, The University of Tokyo, Tokyo, Japan; 2 13143 Department of Gastroenterology, Graduate School of Medicine, The University of Tokyo, Tokyo, Japan; 3 13143 Department of Gastrointestinal Surgery, Graduate School of Medicine, The University of Tokyo, Tokyo, Japan

We present the case of a 52-year-old woman who underwent endoscopic full-thickness resection (EFTR) with one port placement for a 20-mm large gastric submucosal tumor originating from the muscularis propria layer ( Fig. 1 ).

**Fig. 1:**
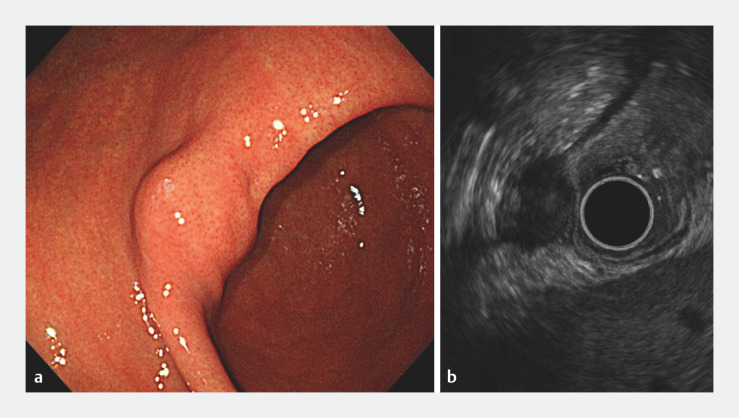
Imaging for the submucosal tumor at the lesser curvature of the gastric angle, originating from the muscularis propria layer. **a** Endoscopic image. **b** Endoscopic ultrasound image.

EFTR was initiated with a mucosal incision around the entire circumference of the lesion using a DualKnife J (KD-655Q; Olympus, Tokyo, Japan), followed by a full-thickness resection using an ITknife2 (KD-611L; Olympus) in combination with traction provided by a multi-loop traction device (Boston Scientific, Marlborough, Massachusetts, USA) 1 .

After resection, we employed novel anchor pronged clips (MANTIS Clip; Boston Scientific) to close the large transmural defect ( Fig. 2 ). The MANTIS Clip was used to grasp one edge of the defect and pull it toward the opposite edge by endoscope manipulation. The anchor prong at the tip of the clip arm prevented the pulled tissue from slipping out when the clip was reopened, allowing it to close over the contralateral edge. We applied three MANTIS Clips for initial closure and reinforced this with re-openable clips to achieve complete closure ( Fig. 3 , Video 1 ). No postoperative complications were observed, and histopathological examination confirmed the diagnosis of schwannoma with R0 resection.

**Fig. 2:**
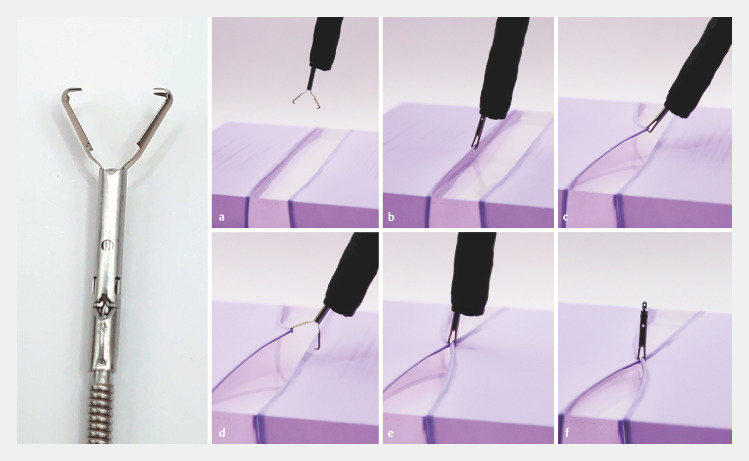
Closure using novel anchor pronged clips (MANTIS Clips; Boston Scientific, Marlborough, Massachusetts, USA). **a–c** The MANTIS Clip is used to grasp one edge of the defect and pull it toward the opposite edge. **d–f** After tissue approximation, the clip is reopened and then closed over the opposite edge before complete deployment.

**Fig. 3:**
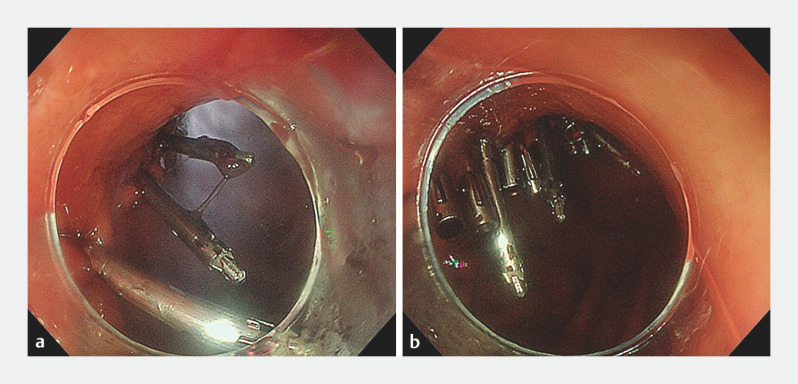
Closure of the defect. **a** The large defect after endoscopic full-thickness resection was initially closed by three MANTIS Clips (Boston Scientific, Marlborough, Massachusetts, USA). **b** Complete closure was achieved by reinforcement with re-openable clips.

Closure of a gastric wall defect after endoscopic full-thickness resection using novel anchor pronged clips.Video 1

While reports exist on the endoloop-assisted closure method 2 and the over-the-scope clip system (OTSC; Ovesco Endoscopy AG, Tübingen, Germany) 3 for defect closure after EFTR, these methods require a dual-channel endoscope, reinsertion of the endoscope, or some special manipulation. OverStitch (Apollo Endosurgery, Austin, Texas, USA) is expected to provide robust full-thickness sutures, but its use requires technical training 4 . The MANTIS Clip, which functions similarly to a conventional through-the-scope clip, offers simple and effective closure with robust grasping force and tissue apposition capability. This novel closure device can be a viable and effective option for defect closure after EFTR.

Endoscopy_UCTN_Code_TTT_1AO_2AI

## Notes

Endoscopy E-Videos https://eref.thieme.de/e-videos
*E-Videos* is an open access online section of the journal *Endoscopy* , reporting on interesting cases and new techniques in gastroenterological endoscopy. All papers include a high-quality video and are published with a Creative Commons CC-BY license. Endoscopy E-Videos qualify for HINARI discounts and waivers and eligibility is automatically checked during the submission process. We grant 100% waivers to articles whose corresponding authors are based in Group A countries and 50% waivers to those who are based in Group B countries as classified by Research4Life (see: https://www.research4life.org/access/eligibility/ ). This section has its own submission website at https://mc.manuscriptcentral.com/e-videos .
